# Birds of a feather? Mis- and dis-information on the social media platform X related to avian influenza

**DOI:** 10.1017/ash.2024.471

**Published:** 2025-01-09

**Authors:** Lauren N. Cooper, Marlon I. Diaz, John J. Hanna, Zachary M. Most, Christoph U. Lehmann, Richard J. Medford

**Affiliations:** 1 Clinical Informatics Center, University of Texas Southwestern Medical Center, Dallas, TX 75390 USA; 2 Paul L. Foster School of Medicine, Texas Tech University Health Sciences Center, El Paso, TX 79430 USA; 3 Division of Infectious Diseases and Geographic Medicine, Department of Internal Medicine, University of Texas Southwestern Medical Center, Dallas, TX 75390 USA; 4 Information Services, ECU Health, Greenville, NC 27834 USA; 5 Brody School of Medicine, Department of Internal Medicine, East Carolina University, Greenville, NC 27834 USA; 6 Department of Pediatrics, University of Texas Southwestern Medical Center, Dallas, TX 75390 USA; 7 Peter O’Donnell School of Public Health, University of Texas Southwestern Medical Center, Dallas, TX 75390 USA

## Abstract

**Objective:**

Social media has become an important tool in monitoring infectious disease outbreaks such as coronavirus disease 2019 and highly pathogenic avian influenza (HPAI). Influenced by the recent announcement of a possible human death from H5N2 avian influenza, we analyzed tweets collected from X (formerly Twitter) to describe the messaging regarding the HPAI outbreak, including mis- and dis-information, concerns, and health education.

**Methods:**

We collected tweets involving keywords relating to HPAI for 5 days (June 04 to June 08, 2024). Using topic modeling, emotion, sentiment, and user demographic analyses, we were able to describe the population and the HPAI-related topics that users discussed.

**Results:**

With an original pool of 14,796 tweets, we analyzed a final data set of 13,319 tweets from 10,421 unique X users, with 50.4% of the tweets exhibiting negative sentiments (< 0 on a scale of −4 to +4). Predominant emotions were anger and fear shown in 36.4% and 29.5% of tweets, respectively. We identified 5 distinct, descriptive topics within the tweets. The use of emotionally charged language and spread of misinformation were substantial.

**Conclusions:**

Mis- and dis-information about the causes of and ways to prevent HPAI infections were common. A large portion of the tweets contained references to a planned epidemic or “plandemic” to influence the upcoming 2024 US presidential election. These tweets were countered by a limited number of tweets discussing infection locations, case reports, and preventive measures. Our study can be used by public health officials and clinicians to influence the discourse on current and future outbreaks.

## Introduction

As news reports of highly pathogenic avian influenza (HPAI) infections in farm animals such as cows and chickens and of transmission to humans increased in the spring of 2024, so did the “bird flu” chatter on social media outlets such as X (formerly Twitter), Instagram, and TikTok. With its proliferation in the 21^st^ century, social media has become an important tool to monitor disease outbreaks and users’ perceptions, allowing public health officials and researchers to determine public sentiment on emergent disease threats and potential interventions.^1^ As seen in recent public health emergencies such as the coronavirus disease 2019 (COVID-19)^2–9^ pandemic and outbreak of mpox,^10,11^ analyzing social media posts on outbreaks can provide vital insight into the public’s response to infection prevention methods such as testing and vaccinations, the handling of infections already present, the spread of mis- and dis-information, and how government and politics play into current and future public health emergencies.

On June 05, 2024, the World Health Organization (WHO) confirmed the first death of a man in Mexico with H5N2 avian influenza infection,^12^ although it was later confirmed that the death was not caused by the H5N2 virus but by existing co-morbidities.^13^ Using this event as a point of reference due to the increase in media coverage, we utilized X to collect tweets focused on the topic of HPAI. By analyzing the tweets using topic modeling, sentiment, and emotion analyses, we hypothesize that we can provide the public health community and government officials useful insights into the fears, concerns, reactions, and possible mis- and dis-information distributed by X users regarding the current HPAI outbreak.

## Methods

### Data collection and preprocessing

We collected all English-language original tweets using keywords indicative of HPAI including the following terms: “H5N1, H5N2, bird flu, bird influenza, avian flu, avian influenza, A(H5N1), A(H5N2), and influenza A virus” using the X application programming interface v2^14^ and the Tweepy Python library v4.14.0.^15^ We chose X as the social media platform of choice due to the availability of numerous Python libraries for the platform as well as the use of short texts, compared to visual media such as images or video of other platforms. The tweets were collected from June 04, 2024, to June 08, 2024, a time span which included the news of the first human fatality associated with HPAI in Mexico along with numerous news outlets and media discussing the ongoing H5N1 HPAI variant outbreak in the United States. Various metadata were also collected such as the author’s username, self-reported location, whether the author was “verified,” the author’s user description, and counts of likes, retweets, impressions, and quotations for each tweet. All data collection, processing, and analyses were conducted using the Python programming language (version 3.10.5).^16^

To prepare the data for analysis, we preprocessed by removing duplicate tweets, embedded URLs, emojis, common symbols such as “#” and “@,” and expanding common contractions. The plain text of the tweets was then cleaned further using the natural language processing library spaCy.^17^ Next, we removed common stop words and created bigrams and trigrams using the Gensim library^18^ to prepare for topic modeling.

## Analyses

### Topic modeling

From the Gensim library, we utilized a latent Dirichlet allocation (LDA) model estimation algorithm to perform topic modeling. Using a corpus based on the trigrams derived from our dataset, we trained LDA models comprising topic numbers from 1 to 40. With U_mass_ coherence scores used to quantitatively determine the optimal number of topics, we ultimately chose a model with 5 topics. Combining the top 20 keywords for each topic with the respective tweets, we utilized OpenAI’s ChatGPT-4^19^ large language model (LLM) to determine appropriate descriptions for each of the 5 topics using the following prompt: “Using the uploaded csv file, can you please give a description of the 5 topics (0-4) using the given keywords, tweets, and percentage of contribution to each topic?”

### Sentiment and emotion analysis

In addition to topic modeling, the sentiment and emotion of the tweets were analyzed to provide additional insight into the public opinions about HPAI. The sentiment of each tweet was determined using the SentiStrength library.^20,21^ With values ranging from −4 for extremely negative sentiments to +4 for extremely positive sentiments, the SentiStrength library is optimized to perform sentiment analysis on short, informal text such as those in tweets and other forms of social media. To determine the emotion present in the tweets, we used the Text2Emotion library,^22^ which uses natural language processing techniques to determine words in the text that express emotion. The text was then categorized, based on probability scores, into 5 emotions: happy, angry, surprise, sadness, and fear.

### User demographics

Although demographics such as age, sex, race, and ethnicity are generally not readily available from tweets, these data can be inferred from user profiles, usernames, profiles, and user images. Using the machine learning-based M3-Inference library,^23,24^ we determined a user’s age range (≤ 18, 19–29, 30–39, ≥ 40), likely binary gender (female or male), and whether a user was “an organization,” meaning the user is likely not an individual person, but rather a corporate entity such as a news organization or company. A user’s ethnicity (Hispanic, non-Hispanic White, non-Hispanic Black, or Asian) was inferred using the Ethnicolr^25^ library. This library uses the user’s first and last name to help determine an accurate ethnicity based on the state of Florida’s voting registration data and US census data.

## Ethical approval

All data included in this study were publicly available and therefore patient consent or approval from an institutional review board were not required.

## Results

### User demographics

During our study period, we collected 14,796 English-language tweets. After preprocessing and the removal of duplicate tweets, we used a final dataset for analyses of 13,319 tweets from 10,421 unique X users. Of the users, 8,150 (77.8%) were individual users and not an organization. Of the users identified as individuals, 5,725 (70.6%) were identified as male, 2,966 (36.6%) were 18 years old or younger, and 2,189 (27%) were 40 years old or older. We were able to identify race in only 84% of individual users (6,846) using the Ethnicolr library, and of those, 78% (5,341) were labeled as non-Hispanic White (Table 1).

**Table 1.** Demographics of the 10,421 unique X users who created the 13,319 tweets from our data set

Demographics	
Is an organization? n (%)	
Not an organization	8,105 (77.8)
Is an organization	2,316 (22.2)
Age n (%) of users identified as not an organization	
18 or under	2,966 (36.6)
19–29	1,296 (16.0)
30–39	1,654 (20.4)
40 or older	2,189 (27.0)
Gender n (%) of users identified as not an organization	
Male	5,725 (70.6)
Female	2,380 (29.4)
Race n (%)^a^	
Non-Hispanic White	5,341 (78.0)
Non-Hispanic Black	625 (9.1)
Asian	533 (7.8)
Hispanic	347 (5.1)

a We were able to identify race in only 6,846 individual users using the Ethnicolr library.

### Sentiment and emotional analyses

**Figure 1.**
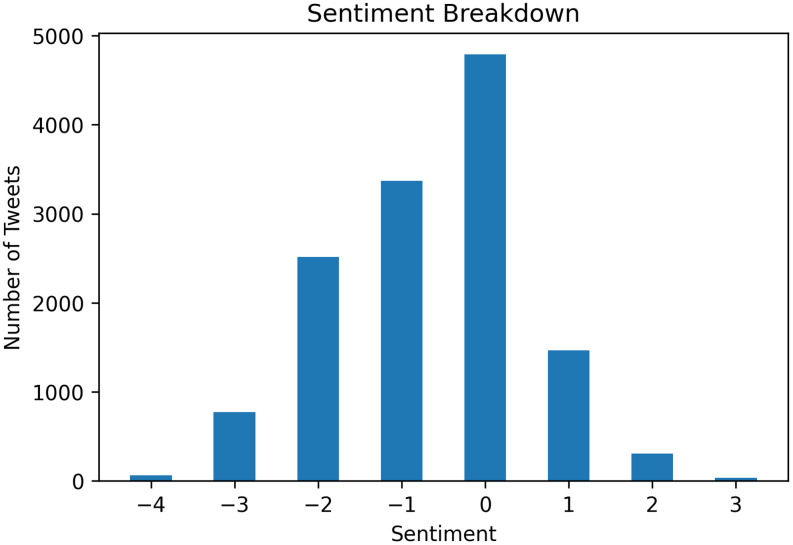
Sentiment analysis of tweets with each sentiment category, ranging from the most negative (−4) to the most positive (+3).

The majority of tweets in our dataset were negative in sentiment. Out of the −4 to +4 scale, 50.4% of the tweets had sentiments less than 0, and the entire data set had a mean sentiment score of −0.68 (1.18). Neutral tweets, those with sentiment scores of 0, accounted for 36.0% of the tweets, while positive tweets (tweets with sentiments greater than 0) only accounted for 13.6% of the dataset (Figure 1).

**Figure 2.**
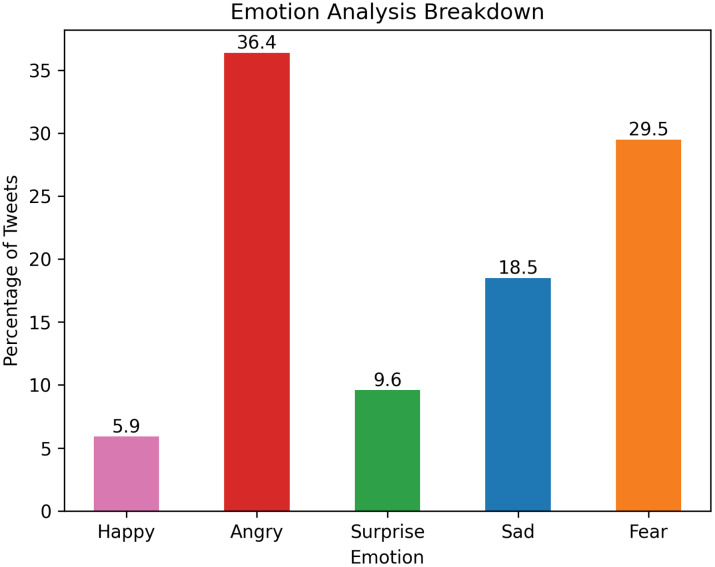
Emotion analysis of tweets based on 5 emotions: happiness, anger, surprise, sadness, and fear.

Of the 5 emotions that could be identified by the Text2Emotion library: happiness, anger, surprise, sadness, and fear, most of the emotions identified in the tweets were negative. Anger (36.4%), fear (29.5%), and sadness (18.5%) comprised most of the tweets exhibited by language such as “F[censored] YOUR BIRD FLU PROPAGANDA,” “Biden et al. are allowing deadly H5N1 to spread unabated! H5N1 has a 50%–60% fatality rate in people-this is catastrophic!,” and “I’m very nervous about H5N1 (and now H5N2) and I’m having the same bad feeling I had before the lockdowns…” In contrast, surprise and happiness made up 9.6% and 5.9%, respectively (Figure 2) as shown by tweets such as “Grateful for health officials in Mexico for transparency,” “Really nice map showing where H5N1 has been detect[ed] in mammals in the U.S.,” and “San Francisco leads with advanced #H5N1 surveillance in wastewater. Commendable efforts!”

### Topic modeling

Utilizing the LDA algorithm and coherence measures, we chose a model with 5 distinct topics (Table 2) to describe the overall themes shown in our data set. Included in these topics was the largest topic containing 3,290 (24.7%) tweets discussing issues with infection controls, spread, and infection reporting as shown by key terms including “confirm,” “infect,” and “spread” and tweets such as “US government enhances protective measures against H5N1 virus in dairy cattle @USDA…” One topic of note (2,326 tweets, 17.5%), entitled “Concerns about Health-Related Misinformation and the Need for Accurate Information,” included key terms such as “scam,” “hoax,” “plandemic,” and “election.” These tweets demonstrated dramatic reactions to the current avian influenza outbreak such as “I wonder when there will be dancing nurses for the upcoming Bird Flu hoax? DO NOT COMPLY” and “BIRD FLU IS NOT A THING! THEY JUST WANT TO TAKE AWAY YOUR FOOD, YOUR FARMS, YOUR STABILITY, YOUR JOBS, YOUR WAY OF LIFE, YOUR FREEDOM!” Other topics in the data set include “personal reactions to a possible avian influenza crisis,” “public sentiment and readiness towards avian influenza,” and “the public’s responses to health measures and policies by health authorities.”

**Table 2.** Topic modeling of tweets. Topic labels were generated by OpenAI’s ChatGPT-4, based on topic keywords, representative tweets, and the percentage of contribution of tweets to the topic model

Topic modeling of tweets			
Topic	Tweets in topic n (%)	Topic keywords	Representative tweets
Infection Control, Spread, and Reporting	3,290 (24.7)	confirm, report, infect, infection, man, spread, person, know, say, animal, world, poultry, state, news, like, detect, cattle, confirm_fatal, kill, risk	• A dairy herd in northwestern Iowa is infected with the H5N1 avian flu virus, said state agriculture secretary Mike Naig on Wednesday. He called on dairy and poultry farmers to harden their biosecurity defenses against the virus. • US government enhances protective measures against H5N1 virus in dairy cattle @USDA, #Global #Africa #foodsafetyafrica #foodsafety #foodbusiness #foodindustry #foodmanufacturing #foodprocessing #foodhygiene #foodquality #regulatory #news #avianinfluenza • Cattle infected with bird flu suffer reduced milk production, digestive issues, fever, and diminished appetite. In South Dakota, a dozen animals sent slaughter after they did not recover from the virus, and killed another dozen that contracted secondary infections (Russ Daly).
Personal Reactions to Possible Avian Influenza Crisis	2,886 (21.7)	come, people, want, f[censored], know, go, comply, s[censored]t, start, day, believe, think, bad, government, get, time, happen, need, report, like	• Don’t listen to anyone trying to scary you with bird flu because it doesn’t exist. People like Deborah Birx have an agenda and that’s to destroy our food supply and to lock us down again and control us. People like that fat f[censored] only care about money and power. • Isn’t this convenient open the border to criminal migrants with all types of diseases now trying to put fear in people f[censored] their fear. f[censored] the bird flu. and f[censored] the W.H.O • THE SAME PEOPLE WHO PULLED OFF THE COV*ID SCAMDEMIC R PULLING OFF THE BIRD FLU SCAMDEMONIC @authorcial its a khazarian takeover and ppl need to WAKE UP. A DAY AFTER ELECTION. Its unBELIEVABLE their GALL these are SOULLESS BEINGS doing this GENOCIDE
Public Sentiment and Public Readiness Towards Avian Influenza	2,440 (18.3)	say, people, time, try, get, like, ready, work, election, go, tell, jab, right, lie, lockdown, think, need, news, worry, fear	• What will you do differently during the upcoming bird flu scam? Will you wear a mask? Stay inside? Take a vax? Id like to hear first hand how you think it should be handled on a personal level. You know its coming, better plan now! • They are relying on short memories, ignorance. and gullibility. Note, they still do not have a test (assay) unique to H5N1. Birx has been publicly embarrassed so many times by her lack of medical knowledge, the WHO CDC need to establish credibility before destroying their cause. • Political careers, votes, & grift matter more to politicians than actual health/safety of people they govern. Do they seriously believe they can outrun consequences of #H5N1 should it become a pandemic? No one is safe until we’re all safe-something those in power fail to realize! • Anyone else worried that current admin will again weaponize national health issue, eg bird flu, into another lockdown just before election? Since dems are better at mail in voting it would give them a great advantage at the polls and would suppress voters who like to vote in
The Public’s Responses to Health Measures and Policies by Health Authorities	2,377 (17.8)	people, go, find, fall, come, way, breaking, let, mrna, cattle, government, good, think, pcr, state, milk, know, lab, happen, time	• USDA Introduces Bulk Milk Testing for Bird Flu in Dairy Cows. The United States Department of Agriculture (USDA) announced a new measure on Thursday allowing farmers to test bulk supplies of milk • Dr. Deborah Birx, former White House COVID response coordinator, wants to PCR test every cow in the United States every week for H5N1 Bird Flu. This flawed plan would likely generate large numbers of false positive H5N1 cases, leading to unnecessary culling of large numbers • Widespread reluctance on the part of farmers to allow scientists government or otherwise onto their premises to study spread of the virus among infected cows has created a frustrating lack of understanding of the dynamics of this outbreak. • I did not expect three things from this #H5N1 discussion: 1) Widespread reluctance by dairy farmers by not allowing scientists to study #H5N1 on their farms. 2) US government is happy to let #H5N1 spread through #dairycow populations. 3) Transmission seems faster than expected.
Concerns About Health-related Misinformation and the Need for Accurate Information	2,326 (17.5)	man, go, kill, time, lie, need, want, public, scam, like, oh, release, plan, hoax, know, come, plandemic, election, let, real	• Since PCR Tests looks like it’s making a comeback for Bird Flu Remember Asymptomatic Symptoms were the main driver of Outbreaks during the Plandemic. Asymptomatic means you’re not showing any symptoms, but have a viral load to pass on to others (bulls[censored]). • NOW HEAR THIS!! BIRD FLU IS NOT A THING! THEY JUST WANT TO TAKE AWAY YOUR FOOD, YOUR FARMS, YOUR STABILITY, YOUR JOBS, YOUR WAY OF LIFE, YOUR FREEDOM! DO NOT COMPLY! #BirdFlu #BirdFluBulls[censored] #DoNotComply #SaveTheChickens #SaveTheCows #SaveTheSun #SaveTheFarmers • Bird Flu = Covid 2024. I Choose Not to Participate. My #Healthy Immune System is far Superior to Any Man Made Drug. Despite the Trend of attempted Unethical Pressure and Coercion, I Refuse to get Any Experimental Jabs or Wear a Useless Face Diaper. I’ll simply use my Brain… • First, it was dancing nurses dancing during the COVID-19 hoax. Then, it was dancing nurses for the climate change hoax. (Featured here) I wonder when there will be dancing nurses for the upcoming Bird Flu hoax? DO NOT COMPLY.

## Discussion

As social media becomes engrained into everyday life, it has become a source of news and information for many. An estimated 50% of US adults receive their news from social media at least some of the time,^26^ the distribution of mis- and dis-information can have devastating effects. Anti-vaccination movements, pseudoscience-based treatments, and inferior infection control efforts for infectious diseases such as COVID-19 and human papillomavirus (HPV) have spread on social media in recent years.^27–29^

In conjunction to the spread of health misinformation, the use of rhetoric such as “DO NOT COMPLY,” “lock us down again,”, the “weaponization” of HPAI, and that the HPAI outbreak is a “hoax”, “plandemic,” or “scamdemic” may be fueled by the upcoming US presidential election. As was shown in the emotion analysis of the tweets, 65.9% of the tweets had evidence of anger or fear. The emotional language used in these tweets provides fodder to further spread the mis- and dis-information using negative tones and expressive language reminiscent of political propaganda. The use of this language aims to discredit information presented by clinicians, public health organizations, and government officials, who hope to protect the public from further harm.

However, social media does have positive effects. During the COVID-19 pandemic, the use of social media was helpful to spread public health information and dispel misinformation.^2^ Additionally, one study demonstrated the use of social media to improve negative opinions on vaccinations for HPV stemming from mis- and dis-information.^30^ Our study did find evidence of the use of the X platform to spread important medical notifications on HPAI infections, news about where the infections are taking place, and measures being taken by health officials to prevent the spread of the disease. This is evident in tweets such as “While the risk of avian influenza infecting #dairy cattle in IL remains low, State Veterinarian Mark Ernst emphasizes the importance of vigilance and biosecurity among farmers,” “USDA: H5N1 now in 80 dairy herds across 9 states,” and “Concerning to see such a large increase in the number of herds infected with #H5N1 #birdflu Re-emphasizing the importance of testing in animals and humans as well as implementing protective measures.” The use of social media, such as X, allows for public health organizations on both global and national levels such as the US Centers for Disease Control and Prevention and the WHO as well as more local organizations such as local health departments and even individual clinician groups to provide the public with fact based, scientifically sound infection prevention and vaccination information in case of further spread of the avian influenza virus.

Our study has several limitations that could limit its ability to be generalized. The first of which is that we collected only English-language tweets because the natural language processing libraries used are trained specifically for English. With the news of the first death associated with the H5N2 variant of HPAI occurring in Mexico, a country in which many speak Spanish, our ability to gain a complete picture of the public’s reaction to this event is limited. Similarly, we did not limit our search to tweets only from Mexico. Location data for tweets are generally poorly collected, with precise locations being turned off in user accounts by default resulting in approximately 1.0% of tweets having a precise geotag and only 30%–40% of tweets having some location information presented in the user profile, according to X documentation.^31^

Additionally, the Ethnicolr library used to identify the race and ethnicities of the X users in the data set was only able to identify the race of 6,846 users, resulting in probable undercounting. This can be caused by a lack of discernable first and last names in the X user’s profile. In 2022, ownership of Twitter changed, and the company was transformed into X. With that, changes in policies and in the number of X users could have affected how representative our data set was to represent the general public. According to recent research by the Pew Research Center, after the transition to X, 20% of US adults on the platform create approximately 98% of all tweets,^32^ indicating that only a small portion of the public opinion is represented by this platform.

Through the analyses of over 13,000 tweets discussing the HPAI outbreak in the spring of 2024, we were able to show that a large portion of the tweets represented feelings of anger and fear or included the dissemination of mis- and dis-information about a “plandemic” and urged others “Do Not Comply” with the information presented by government officials about the outbreak. A bright spot in the analyses did show that information important to public health was also presented on X via tweets indicating locations of outbreaks among dairy and poultry farms and reminders urging workers to use preventative measures when working near affected livestock. Future monitoring of social media posts regarding outbreaks of infectious diseases will further deepen our knowledge of how the public will and does respond to public health emergencies.

## Research Transparency and Reproducibility

Due to restrictions from X Corp and the X Developers license, the availability of our data set is limited. Tweet IDs/user IDs are available from the authors with written permission from X Corp.
